# Paternal Tobacco Smoke Correlated to Offspring Asthma and Prenatal Epigenetic Programming

**DOI:** 10.3389/fgene.2019.00471

**Published:** 2019-05-31

**Authors:** Chih-Chiang Wu, Te-Yao Hsu, Jen-Chieh Chang, Chia-Yu Ou, Ho-Chang Kuo, Chieh-An Liu, Chih-Lu Wang, Hau Chuang, Chie-Pein Chen, Kuender D. Yang

**Affiliations:** ^1^ Department of Pediatrics, Po-Zen Hospital, Kaohsiung, Taiwan; ^2^ Institute of Clinical Medicine, National Yang-Ming University, Taipei, Taiwan; ^3^ Department of Obstetrics and Gynecology, Kaohsiung Chang Gung Memorial Hospital, Chang Gung University College of Medicine, Kaohsiung, Taiwan; ^4^ Genomic and Proteomic Core Laboratory, Department of Medical Research, Kaohsiung Chang Gung Memorial Hospital, Chang Gung University College of Medicine, Kaohsiung, Taiwan; ^5^ Department of Obstetrics, Po-Zen Hospital, Kaohsiung, Taiwan; ^6^ Department of Pediatrics, Kaohsiung Chang Gung Memorial Hospital, Chang Gung University College of Medicine, Kaohsiung, Taiwan; ^7^ Department of Obstetrics and Gynecology, Mackay Memorial Hospital, Taipei, Taiwan; ^8^ Department of Pediatrics, Mackay Memorial Hospital, Taipei, Taiwan; ^9^ Institute of Biomedical Sciences, Mackay Medical College, New Taipei City, Taiwan; ^10^ Institute of Microbiology and Immunology, National Defense Medical Center, Taipei, Taiwan

**Keywords:** paternal tobacco smoke, prenatal tobacco smoke exposure, asthma development, CG methylation, *LMO2*, *IL-10*, *GSTM1*

## Abstract

**Rationale:** Little is known about effects of paternal tobacco smoke (PTS) on the offspring’s asthma and its prenatal epigenetic programming.

**Objective:** To investigate whether PTS exposure was associated with the offspring’s asthma and correlated to epigenetic CG methylation of potential tobacco-related immune genes: *LMO2, GSTM1* or/and *IL-10* genes.

**Measurements and Main Results:** In a birth cohort of 1,629 newborns, we measured exposure rates of PTS (23%) and maternal tobacco smoke (MTS, 0.2%), cord blood DNA methylation, infant respiratory tract infection, childhood DNA methylation, and childhood allergic diseases. Infants with prenatal PTS exposure had a significantly higher risk of asthma by the age of 6 than those without (*p* = 0.026). The PTS exposure doses at 0, <20, and ≧20 cigarettes per day were significantly associated with the trend of childhood asthma and the increase of *LMO2-E148* (*p* = 0.006), and *IL10_P325* (*p* = 0.008) *CG* methylation. The combination of higher CG methylation levels of *LMO2_E148, IL10_P325*, and *GSTM1_P266* corresponded to the highest risk of asthma by 43.48%, compared to other combinations (16.67–23.08%) in the 3-way multi-factor dimensionality reduction (MDR) analysis. The *LMO2_P794* and *GSTM1_P266* CG methylation levels at age 0 were significantly correlated to those at age of 6.

**Conclusions:** Prenatal PTS exposure increases CG methylation contents of immune genes, such as *LMO2* and *IL-10*, which significantly retained from newborn stage to 6 years of age and correlated to development of childhood asthma. Modulation of the *LMO2* and *IL-10* CG methylation and/or their gene expression may provide a regimen for early prevention of PTS-associated childhood asthma.

**Descriptor number:** 1.10 Asthma Mediators.

**Scientific Knowledge on the Subject**: It has been better known that maternal tobacco smoke (MTS) has an impact on the offspring’s asthma *via* epigenetic modification. Little is known about effects of paternal tobacco smoke (PTS) on the offspring’s asthma and its prenatal epigenetic programming.

**What This Study Adds to the Field**: Prenatal tobacco smoke (PTS) can program epigenetic modifications in certain genes, such as *LMO2* and *IL-10*, and that these modifications are correlated to childhood asthma development. The higher the PTS exposure dose the higher the CG methylation levels are found. The combination of higher CG methylation levels of *LMO2_E148, IL10_P325* and *GSTM1_P266* corresponded to the highest risk of asthma. Measuring the DNA methylation levels of certain genes might help to predict high-risk populations for childhood asthma and provide a potential target to prevent the development of childhood asthma.

## Introduction

The prevalence of childhood asthma has increased worldwide in recent decades. A complex disease such as hypertension or asthma is generally considered to be programmable by specific types of early-life environmental exposure (Barker, 2001; Brew et al., 2017). Early life exposure to environmental factors such as nutritional deprivation during pregnancy has been shown to increase the risk of low birth weight in offspring (Haggarty et al., 2009). In addition to low birth weight, early life exposure to air pollution (Schultz et al., 2016), or maternal tobacco exposure (MTS) (Gilliland et al., 2001; Wang et al., 2002; Sharma, 2017) has also been shown to increase the risk of childhood asthma. Maternal smoking during pregnancy increased the occurrence of physician-diagnosed asthma and wheezing during childhood, (Gilliland et al., 2001) and maternal exposure of particulate matter air pollution during pregnancy gave birth to newborns with significantly lower telomere length, a marker of biological aging that may provide a cellular memory of exposures to oxidative stress and inflammation (Martens et al., 2017), suggesting maternal exposure in prenatal period is a critical window for cellular programming of asthma development. It is not clear whether paternal tobacco smoke (PTS) could increase the risk of asthma in the offspring.

A number of environmental factors related to asthma development have been reported to be associated with differential DNA methylation (Perera et al., 2009; Tarantini et al., 2009; Nadeau et al., 2010; Shenker et al., 2013; Ivorra et al., 2015). Perera et al. (2009) found that methylation of the acyl-CoA synthetase long-chain family member 3 (*ACSL3*) promoter in umbilical cord blood leukocytes was significantly associated with maternal airborne polycyclic aromatic hydrocarbon (PAH) exposure and with parent-reported asthma symptoms in children prior to age of 5. Increased CG methylation of the *FOXP3* gene has been reported in Treg DNA from asthmatic children who exposed to high levels of ambient air pollution compared to a low-exposure group and was associated with increased asthma morbidity (Nadeau et al., 2010). The differential methylation changes of four genomic loci (AHRR, 6p21, and two at 2q37) have been reported to have high positive prediction and sensitivity values for predicting previous smoking status (Shenker et al., 2013). All of these studies suggest a possible epigenetic mechanism underlying the life-long effect of prenatal environmental exposure including MTS on altered immune regulation and development of asthma.

Our previous studies have shown that effects of gene-gene and gene-environment interactions on IgE production begin in the prenatal stage, female children with null genotype of *GSTM1* are more susceptible to tobacco-associated asthma, and male children of non-atopic parents living without air filters had a higher risk to asthma (Liu et al., 2003; Yang et al., 2010; Wu et al., 2014; Lee et al., 2017). We also found that the rate of MTS (<1%) was much lower than that of PTS (21%) in the Taiwanese birth cohort (Liu et al., 2003), rendering the opportunity to study the impact of PTS on the offspring’s childhood asthma. Recently, DNA methylation patterns in neonates with early-life exposure of pollutants have partly been deciphered (Hobbs et al., 2014; Menezo et al., 2015; Rotroff et al., 2016). Maternal tobacco smoke impacts CG methylation program in certain biological pathways including T lymphocyte regulatory genes (Rotroff et al., 2016). Prenatal tobacco exposure has a significant impact on asthma development and DNA methylation dependent on the polymorphism of the redox gene, *GSTM1* (Breton et al., 2009; Rogers et al., 2009; Wu et al., 2014). Therefore, we postulated that prenatal exposure of PTS might program epigenetic modifications of immune or/and genotoxicant detoxification genes, which could be programmed in utero, retained into childhood, and contributed to the development of childhood asthma. It is hoped that clarifying the prenatal epigenetic program of immune and detoxification genes for asthma development may provide potentially reversal strategies of DNA methylation for the early prediction and prevention of PTS-associated asthma.

## Materials and Methods

### Study Design and Subjects

A longitudinal birth cohort study involving 1,629 newborns was conducted at Kaohsiung Chang Gung Memorial Hospital, Taiwan. The study protocol was approved by the Institutional Review Board of Chang Gung Memorial Hospital, Taiwan. All parents signed a written informed consent form after being informed of the study protocol and were requested to follow up with their children. Among the 1,629 newborns born in the study hospital, 1,348 and 756 children completed the 18-month and 6-year follow up, respectively (Wu et al., 2014; Lee et al., 2017). The information about the parental atopic history and family smoking habits, including the daily amount of cigarette consumption in each family, was obtained from parents during prenatal recruitment. Twenty-three percent of the fathers (367/1629) reported tobacco smoke but only three mothers (0.2%) reported to have tobacco smoke in this cohort. This unique culture character in this birth cohort is therefore suitable for studying effects of paternal tobacco smoke exposure (PTS) on the offspring infant (age of 18 months) upper respiratory tract infections (URIs) and childhood (age of 6) asthma.

Cord blood samples were collected immediately after the infant’s birth for measuring the IgE levels using the Pharmacia CAP system (Uppsala, Sweden) and DNA collection. Blood samples were collected from the children at follow-up of both 18 months and 6 years of age to measure total IgE levels and allergen sensitization based on detectable specific IgE levels in response to egg white (f1), cow’s milk (f2), peanuts (f13), shrimp (f24), house dust mites (d1), and the German cockroach (i6) (Phadia CAP system), as house dust mites (approximately 90%) and German cockroaches (15–42%), rather than pollen or mold (both <2%), are the major aeroallergens for children with asthma in Taiwan (Lin et al., 2002; Huang et al., 2006; Wan et al., 2010). In the 18-month follow up, eczema history, frequency of URIs (≤2, 3–4, and > 4 times), and wheezing episodes were collected *via* a questionnaire. In the 6-year follow up, the atopic diseases of the children, including atopic dermatitis, allergic rhinitis or asthma that had ever been diagnosed by a physician were acquired.

### Preparation of DNA Samples

In previous genetic association studies, umbilical cord blood leukocytes were subjected to DNA extraction using the Gentra Purgene kit (Qiagen Inc., Valencia, CA), stored in DEPC-treated water at −80°C following 70% alcohol precipitation and used for genetic association analyses (Liu et al., 2003; Yang et al., 2010; Wu et al., 2014). In this study, we searched for the quality and quantity of the DNA samples from the stock and found 361 cord blood DNA samples available for CG methylation assay. Of the 361 subjects with cord blood DNA samples, 211 had the DNA samples of children at 6 years of age qualified for the matched analysis on the changes of CG methylation contents in prenatal and postnatal stages.

### Screening of DNA CG Methylation Profiles Between Newborns with and Without PTS Exposure

Methylation detection with Illumina GoldenGate methylation Panel I array assay for 1,505 CG sites was performed as manufacturer’s direction. Briefly, for each CG site, four probes were designed: two allele-specific oligos (ASO) and two locus-specific oligos (LSO). Each ASO-LSO oligo pair corresponded to either the methylated or unmethylated state of the CpG site. Bisulfite conversion of DNA samples was done using the EZ DNA methylation kit (Zymo Research Co., Orange, CA). For each experiment, 5 μl bisulfite-converted DNA sample (for each 250 ng before conversion) was used. After bisulfite treatment, the remaining assay steps were identical to the genotyping array. The array hybridization was conducted under a temperature gradient program and imaged using a BeadArray Reader scanner. Image processing and intensity data extraction software were Illumina BeadScan (version 3.5.49) and Illumina BeadStudio (version 3.1.3.0) with Methylation Analysis Module (version 3.2.0). Technical replicates of each bisulfite-converted sample were run. The duplicates all agreed well with each other (average r2 = 0.98) and were averaged together for further analysis.

### Validation of CG Site Methylation Levels Through Bisulfite Pyrosequencing

The bisulfite pyrosequencing method was used to detect the methylation levels of promoter or exon one CG sites of genes studied. Pyrosequencing was performed using the PyroMark Q24 Pyrosequencing System (Qiagen, Valencia, CA, USA) (Colella et al., 2003). The primer sequences and annealing temperatures of the pyrosequencing of *LMO2_E148, LMO2_P794, GSTM1_P266*, and *IL10_P325* are listed below. *LMO2_E148* CG methylation (cg22902574) levels were measured by forward primer 5′-GGGTAGGTGGGGGTATTTTT-3′, reverse primer 5′-CTAAAAATCACAAATCTCCACA-3′, and sequencing primer 5′-TGGTTGTTTATTTGATAGGG-3′ with annealing temperature 59°C; *LMO2_P794* CG methylation (cg02028355) levels were measured by forward primer 5′-GAGTTTTGAAGTTTTGGTTTGTAATTTG-3′, reverse primer 5′-CTCTTATACTCAAACCACCACC-3′, and sequencing primer 5′-GTTTTTGTTTGTTAGTTATT-3′ with annealing temperature 66°C; *GSTM1_P266* CG methylation (cg19763514) levels were measured by forward primer 5′-TATAGTGATAGGGGTTGAATTAA-3′, reverse primer 5′-CTACCCAACCCTAAAACTCC-3′, and sequencing primer 5′-GATTTGGTTGGTGTTTTAAG-3′ with annealing temperature 59°C; *IL-10_P325* CG methylation [chromosome 1, position 205,012,787 in hg18 (NCBI 36.1)] levels were measured by forward primer 5′-TGTAAGTTTAGGGAGGTTTTTTTAT-3′, reverse primer 5′-CCCAATTATTTCTCAATCCCATTATATTC-3′, and sequencing primer 5′-GAGGTTTTTAGTTGTGG-3′ with annealing temperature 60°C. All the DNA methylation contents of these CG sites validated by the bisulfite pyrosequencing method, which disclosed the methylation contents of 3–5 CG sites in a primer set, showed highly significant linkage of CG methylation contents among the CG sites studied (*p* < 0.001).

### Data Analysis and Statistics

The demographic data from children with and without prenatal PTS exposure were analyzed with Chi-Squared tests, as previously described (Liu et al., 2003; Yang et al., 2010; Wu et al., 2014; Lee et al., 2017). The log transformed IgE levels at 18 months and 6 years of age were analyzed with Student *t*-test. The CG methylation levels in cord blood from newborns with tobacco smoke exposure less than 20 cigarettes per day, those with tobacco smoke exposure more than 20 cigarettes per day, and those without PTS exposure were analyzed through one-way ANOVA and *post-hoc* analysis using the LSD method. The study power of the sample size at 361 for the comparison of CG methylation contents between children with and without prenatal PTS exposure is 0.9, based on an alpha level of 0.05 and an effect size of 0.10 (10% difference of CG methylation content). The study power of the sample size at 211 pairs of the newborn (0 year) and children (6 years) matched DNA samples for comparing the changes of CG methylation contents between newborns and children is 0.8, based on an alpha level of 0.05 and an effect size of 0.10. The interactions of the higher or lower CG methylation levels among *LMO2, IL10*, and *GSTM1* cut-off by receiver operating characteristic (ROC) curve were analyzed in relation to the development of childhood asthma using multi-factor dimensionality reduction software (MDR 2.0 beta 6, open-source software accessible at http://sourceforge.net/projects/mdr/), which reduced the number of multi-factorial dimensions of two-way (Ritchie et al., 2003), and enabled high- and low-risk classifications to be made regarding the role of gene-gene interactions in childhood asthma (Yang et al., 2010).

## Results

### Demographic Data on the Birth Cohort Population with and Without Completion of Follow-Up and with and Without Paired DNA Samples Available for Analysis

This study followed up 1,629 newborns born in the study hospital. Among the 1,629 newborns, 1,348 infants completed the 18-month follow-up and 756 children completed the follow up at 6 years of age. As described previously, demographic data were not significant differences between the children with completion and incompletion of the 6-year cohort follow-up (Wu et al., 2014; Lee et al., 2017). To investigate the correlation of prenatal PTS exposure to the cord blood DNA CG methylation content, demographic data on types of delivery, gestational age, prematurity, gender, levels of umbilical cord blood IgE, and parental allergic diseases and sensitization were not significant differences between those with and without qualified DNA samples (*p* > 0.05).

### Prenatal Paternal Smoke Exposure Associated with Infant Frequent URIs and Childhood Asthma

In the prenatal questionnaire analysis, we found that the exposure rates of prenatal PTS and MTS were, respectively, 23.0 and 0.2% in this birth cohort. Prenatal PTS exposure was not significantly associated with infant eczema (20.9 vs. 24.0%, *p* = 0.267) or infant wheezing (10.6 vs. 13.8%, *p* = 0.157). In the follow up at age of 18 months, we found that the rates of frequent URIs (>4 times) and non-frequent URIs (≤4 times) were, respectively, 63.9 and 31.9%. The frequent URIs in infants with prenatal PTS exposure were significantly higher than those without prenatal PTS exposure (70.2 vs. 61.6%, *p* = 0.007). Children with prenatal PTS exposure had a significantly higher risk of asthma than those without (30.9 vs. 22.8%, *p* = 0.026). However, there were no significant differences of asthma rates in infant or childhood total IgE levels, or allergic sensitization of children at the age of 6 (Table 1). The prenatal PTS exposure was not significantly associated with the development of atopic dermatitis (29.8 vs. 32.9%, *p* = 0.442) or rhinitis (57.1 vs. 55.1%, *p* = 0.639).

**Table 1:** Allergic sensitization and diseases in children with and without prenatal PTS.

	Prenatal TSE		No TSE		*p*
Cord blood IgE (≥0.35 kU/L)	19.30%	59/306	20.70%	214/1036	0.600
18 m eczema	20.90%	63/301	24.00%	239/995	0.267
18 m URI > 4 times	70.20%	207/295	61.60%	597/969	0.007
18 m wheezing	10.60%	32/301	13.80%	137/995	0.157
6 y HDM sensitization^*^	46.50%	87/187	47.00%	255/544	0.918
6 y food allergen sensitization^#^	26.20%	49/187	23.30%	127/544	0.43
6 y any sensitization^〒^	54.50%	102/187	52.00%	283/544	0.551
6 y atopic dermatitis	29.80%	57/191	32.90%	183/557	0.442
6 y rhinitis	57.10%	109/191	55.10%	307/557	0.639
6 y asthma	30.90%	59/191	22.80%	127/557	0.026
Log transformed 18 m IgE^@^	1.74 ± 0.04		1.69 ± 0.02		0.186
Log transformed 6y IgE^@^	1.87 ± 0.05		1.87 ± 0.03		0.944

* HDM sensitization is defined by specific IgE against house dust mites (d1) ≧0.35 kU/L.

# *Food allergen sensitization is defined by detectable serum levels of specific IgE (≥0.35 kU/L) against one or more food allergens (egg white, cow’s milk, peanuts, and shrimp)*.

〒 *Any sensitization is defined by detectable serum levels of specific IgE (≥0.35 kU/L) against one or more common allergens (egg white, cow’s milk, peanuts, shrimp, house dust mites, and the German cockroach)*.

@ *Log transformed 18 m and 6 y IgE is represented as mean ± standard error mean*.

### Genes with 10% Higher and Lower CG Methylation Contents in Newborns with Prenatal PTS

**Figure 1:**
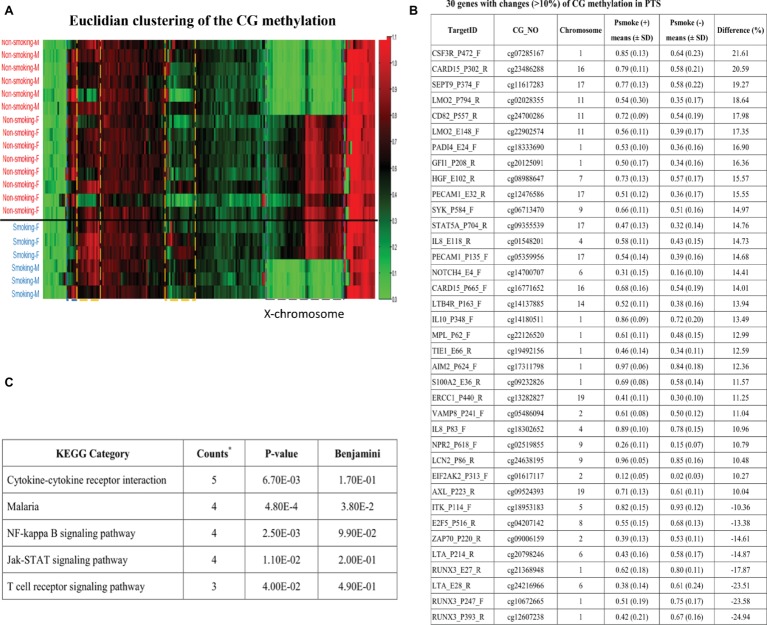
Euclidian clustering of the CG methylation profiles between newborns with and without PTS. **(A)** Female (F) X-chromosome revealed one half of the CG sites were methylated (red color) but not male (M) ones (green), and there are other gene clusters associated with prenatal exposure of PTS (data deposited to the GEO Repository at accession number GSE129751); **(B)** Thirty-seven CG sites in 30 genes had 10% higher or lower CG methylated contents, in which 29 CG sites were higher and 8 CG sites were lower CG methylation; **(C)** The KEGG pathway analysis found five pathways including cytokine-cytokine receptor interaction, malaria defense, NF-kappa B signaling, Jak-STAT signaling, and T cell receptor signaling pathways were significantly involved in CG methylation changes associated with prenatal exposure of PTS. The five genes in cytokine-cytokine receptor interaction are IL8, MPL, CSF3R, IL10, and LTA; the four genes in Malaria defense are IL8, HGF, IL10, and PECAM1; the four genes in NF-kappa B signaling pathway are IL8, LTA, SYK, and ZAP70; the four genes in Jak-STAT signaling pathway are MPL, CSF3R, IL10 and STAT5A; and the three genes in T cell receptor signaling pathway are ITK, IL10, and ZAP70.

To investigate whether PTS was associated with prenatal epigenetic programming of CG site methylation, 20 cord blood DNA samples from newborns with and without PTS were subjected to microarray assay of 1,505 CG loci in 807 genes by GoldenGate^®^ (Illumina, Inc., San Diego, CA) methylation bead system. In an algorithm by Euclidian clustering metric analysis, DNA methylation of CG sites in the female X-chromosome was demonstrated by one half of CG sites with methylation (Figure 1A), indicating that CG site methylation array could be an efficient tool to identify CG site methylation content of genes in human chromosomes. The Euclidian clustering also identified other gene clusters with higher or lower of CG site methylation contents different between newborns with and without prenatal exposure of PTS (data are deposited to the GEO Repository at accession number GSE129751). While cutting off the 10% higher or lower of CG methylation contents between newborns with and without PTS, we found 37 CG sites in 30 genes revealed more than 10% difference, in which 29 CG sites had higher CG methylation and 8 CG sites had lower CG methylation (Figure 1B). KEGG pathway analysis showed five pathways including cytokine-cytokine receptor interaction, malaria defense, NF-kappa B signaling, and Jak–STAT signaling, and T cell receptor signaling pathways were significantly involved in the CG methylation change associated with prenatal exposure of PTS (Figure 1C).

### Validation and Correlation of *LMO2_P794* and *LMO2_E148* and *IL10_P325* but not *GSTM1_P266 CG* Methylation Levels with Prenatal PTS and Childhood Asthma

**Figure 2:**
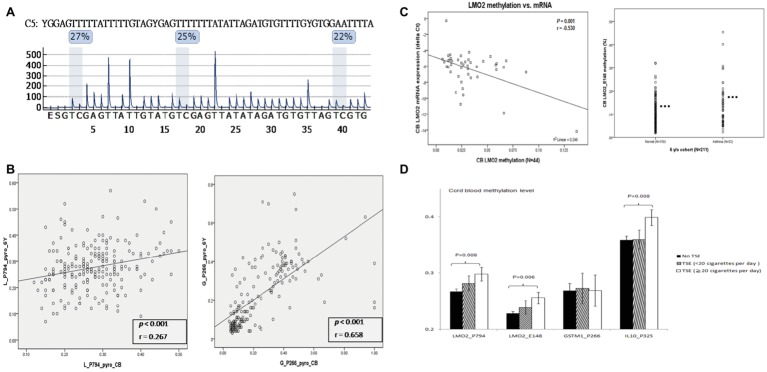
Quantitative and functional validation of *LMO2* methylation contents in different methods, ages, mRNA expression, and childhood asthma. **(A)** Quantification of CG methylation by pyrosequencing. The difference of methylated and unmethylated *LMO2* promoter CG sites across the P794 region was located between 22 and 27%. **(B)** The LMO2_P794 CG methylations levels in the newborn stage (X-axis) revealed a significant correlation to those at 6 years of age (Y-axis) (*r* = 0.267, *p* < 0.001), and GSTM1_P266 CG methylation levels at the newborn stage were highly significantly correlated with those at 6 years of age (*r* = 0.658, *p* < 0.001). The statistics is analyzed by Pearson coefficiency. **(C)** Functional association of LMO2 CG methylation contents with mRNA expression and childhood asthma. The higher the CG methylation contents the lower mRNA expression (left panel). The statistics is analyzed by Pearson coefficiency. In the student *t*test, children with asthma had a significantly higher CG contents than those without asthma (right panel). **(D)** *LMO2_P794, LMO2_E148,* and *IL10_P325, but not GSTM1_P266* CG sites revealed significantly higher CG methylation contents in those with a heavy prenatal exposure of PTS ≥ 20 cigarettes/day (black: no smoke; slash: <20 cigarettes/day; white: ≥20 cigarettes/day). The statistics is analyzed by one-way ANOVA and *post-hoc* test.

To validate the association of higher CG methylation contents in these CG methylation candidate genes with prenatal PTS exposure or childhood asthma, we subjected qualified cord blood DNA samples from 361 patients of the 756 children for measurement of the CG site methylation levels of *LMO2, GSTM1*, and *IL10* by pyrosequencing. As shown in Figure 2A, we found that the difference of *LMO2* promoter CG contents between methylated and unmethylated contents ranged between 22 and 27% in pyrosequencing assay (Figure 2A), in comparison to the data of 18.6% in microarray assay (Figure 1B). A receiver operating characteristic (ROC) curve was used to determine the optimal cut-off levels of methylation at these CG sites to predict childhood asthma. The results showed that *LMO2_E148* CG and *LMO2_P794* methylation levels higher than 28.5 and 29.5% at the newborn stage indicated a significantly higher risk of developing asthma (*p* = 0.003 and *p* = 0.05, respectively), whereas *IL10_P325 CG* methylation level higher than 38.5% showed borderline significance (*p* = 0.067) in the association of asthma development; there was no significant difference on the asthma development between *GSTM1_P266 CG* methylation levels higher and lower than 41.5% (Table 2).

**Table 2:** Prediction of asthma by cutting off CG site methylation levels of *LMO2*, *GSTM1*, and *IL10* according to the ROC curve followed by DeLong test.

CG sites	Cuff-off levels	*p*	OR (95% CI)
*LMO2_P794*	0.295	0.05	1.630 (0.999–2.660)
*LMO2_E148*	0.285	0.003	2.204 (1.299–3.741)
*IL10_P325*	0.385	0.067	1.576 (0.967–2.566)
*GSTM1_P266*	0.415	0.875	1.046 (0.599–1.824)

Further validation disclosed that the *LMO2_P794, LMO2_E148*, and *IL10_P325* methylation levels detected in cord blood were significantly higher in subjects with prenatal PTS exposure than in those without exposure (*p* = 0.005, 0.009, and 0.033, respectively) (Table 3), and in subjects with physician-diagnosed asthma than those without asthma (*p* = 0.019, 0.003, and 0.076, respectively) in univariate analysis (Table 3). Interestingly, the *IL10_P325* CG methylation levels were significantly associated with prematurity (gestational age < 37 weeks) in univariate analysis (Table 3). While personal and environmental factors as well as the allergic disease phenotypes recorded at 6 years of age were included in the multifactor logistic regression analysis, the CG methylation levels of *LMO2_P794* and *LMO2_E148*, but not *GSTM1_P266* or *IL10_P325*, were significantly associated with prenatal PTS exposure and childhood asthma (Table 3). In multivariate analysis, prematurity also appeared to have a significantly lower methylation content of *IL10_P325 CG* (*p* = 0.017).

**Table 3:** Correlation of the CpG methylation levels of *LMO2_P794*, *LMO2_E148*, *GSTM1_P266*, and *IL10_P325* to prenatal PTS and allergic diseases.

Prenatal factors	LMO2_P794		LMO2_E148		GSTM1_P266		IL10_P325	
	UVA^*^	MVA^#^	UVA^*^	MVA^#^	UVA^*^	MVA^#^	UVA^*^	MVA^#^
Prenatal PTS	0.005	0.026	0.009	0.046	0.916	0.969	0.033	0.129
CBIgE≧0.5kU/L	0.080	0.164	0.456	0.933	0.920	0.836	0.354	0.938
Maternal atopy	0.110	0.463	0.262	0.647	0.732	0.817	0.028	0.094
Paternal atopy	0.837	0.782	0.524	0.896	0.280	0.178	0.625	0.422
Gender (male)	0.410	0.915	0.049	0.363	0.895	0.714	0.011	0.178
Preterm (<37 weeks)	0.629	0.759	0.983	0.918	0.395	0.407	0.030	0.013
18 m eczema	0.119	0.396	0.712	0.600	0.543	0.423	0.957	0.681
18 m URI > 4 times	0.393	0.383	0.213	0.289	0.713	0.883	0.843	0.980
18 m wheezing	0.433	0.368	0.972	0.877	0.987	0.736	0.467	0.552
6 y asthma	0.019	0.019	0.003	0.005	0.860	0.941	0.076	0.216
6 y rhinitis	0.389	0.088	0.271	0.015	0.646	0.480	0.898	0.204
6 y dermatitis	0.314	0.294	0.580	0.236	0.485	0.782	0.948	0.601

*Notes: M, month-old; y, year-old*.

* *UVA: Univariate linear regression analysis*.

# *MVA: Multivariate linear regression analysis; CBIgE, cord blood immunoglobulin E; URI, upper respiratory tract infection*.

### Functional Correlations of the PTS-Associated CG Methylation Contents to Age (From the Newborn Stage to 6 Years of Age), mRNA Expression, and Childhood Asthma

Paired DNA samples collected at 0 and 6 years of age in 211 children were subjected to measurement of *LMO2_P794* and *GSTM1_P266* CG site methylation levels. The results showed that the *LMO2_P794* CG methylation levels in the newborn stage revealed a significant correlation to those at 6 years of age (Figure 2B, left panel: *r* = 0.267, *p* < 0.001), and *GSTM1_P266* CG methylation levels at the newborn stage were highly significantly correlated with those at 6 years of age (Figure 2B, right panel: *r* = 0.658, *p* < 0.001). Based on the higher and lower correlation coefficiency (*r* = 0.658 vs. *r* = 0.244), it appeared that *GSTM1_P266* CG methylation content was prominently determined in prenatal sage, and the *LMO2_P794* CG methylation content was determined both in prenatal and postnatal stages. We also found the higher the *LMO2_P794* CG methylation contents the lower *LMO2* mRNA expression in 44 paired samples (Figure 2C, left panel; *p* < 0.001, *r* = 0.53); children with asthma at 6 years of age had a significantly higher CG methylation content of *LMO2* than those without (Figure 2C, right panel; *p* < 0.005, *n* = 211). Nevertheless, children with prenatal PTS exposure corresponding to more than 20 cigarettes per day had a significantly higher risk of developing asthma than those with less than 20 cigarettes per day and those without prenatal PTS exposure (35, 25, 22.7%, respectively, *p* = 0.033). The prenatal PTS exposure doses (0, less than 20, more than 20 cigarettes per day) also correlated to CG methylation contents of *LMO2_P794* (*p* = 0.008)*, LMO2_E148* (*p* = 0.006), and *IL10_P325* (*p* = 0.008) (Figure 2D).

### Prediction of Childhood Allergic Diseases Based on the Interaction Between High/Low CG Methylation Levels of the Genes Studied

**Figure 3:**
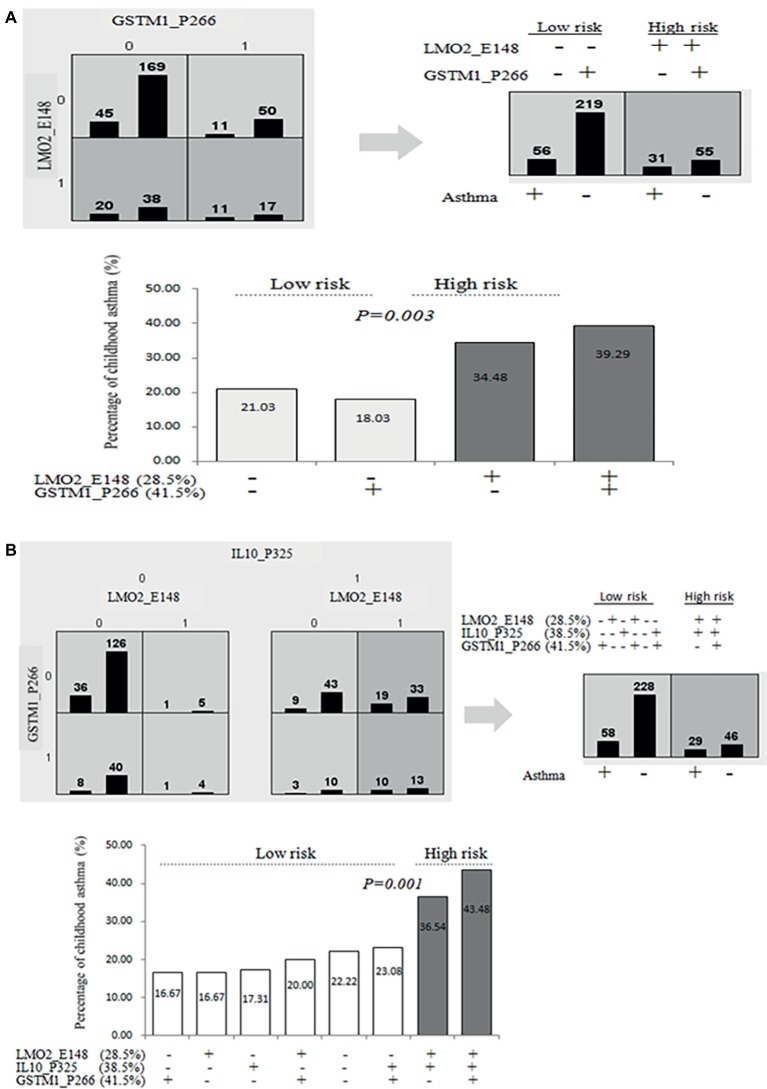
Prediction of asthma by combined higher methylation contents of *LMO2_P794, LMO2_E148, GSTM1_P266* or/and *IL10_P325* CG sites. **(A)** Prediction of higher or lower *LMO2_E148, IL10_P325*, and *GSTM1_P266* CG methylation levels (based on ROC curve) for childhood asthma determined by MDR analyses in two-way mode; **(B)** prediction of higher *LMO2_E148, IL10_P325*, and *GSTM1_P266* CG methylation levels (based on ROC curve) for childhood asthma determined by MDR analyses in three-way mode.

Based on the cut-off CG methylation levels of ROC curve for the asthma prediction, we further used multifactor dimensionality reduction (MDR) assay to identify that the combinations of higher CG methylation levels of *LMO2_E148* (≧28.5%) and *GSTM1_P266* (≧41.5%), and higher CG methylation levels of *LMO2_E148* and *IL10_P325* (≧38.5%) predicted a significantly higher risk of childhood asthma (Table 4). The combination of higher *LMO2_E148* and *GSTM1_P266* CG methylation levels predicted the highest rate of asthma risk (39.29%), compared to other combinations of both or either one CG methylation levels (*p* = 0.003, OR: 2.20, 95% CI: 1.30–3.74) (Figure 3A). The combination of higher *LMO2_E148*, *IL10_P325* and *GSTM1_P266* methylation levels corresponded to the highest risk of asthma 43.48%, compared to the low-risk combinations (16.67–23.08%) (*p* = 0.001, OR: 2.48, 95% CI: 1.43–4.28) (Figure 3B).

**Table 4:** Interactions among genes with higher CG contents in relation to asthma diagnosed at 6 years of age in MDR analyses.

Rank*	Gene-gene interaction		Balanced accuracy (%)^#^	*p* ^§^
**1) Two-way interactions**				
1	*LMO2_P794*	*LMO2_E148*	0.5788	0.2517
2	*LMO2_P794*	*IL10_P325*	0.5636	0.0577
3	*LMO2_794*	*GSTM1_P266*	0.5584	0.0207
4	*LMO2_E148*	*IL10_P325*	0.5828	0.0059
5	*LMO2_E148*	*GSTM1_P266*	0.5778	0.0207
6	*IL10_P325*	*GSTM1_P266*	0.5550	0.0577
**2) Three-way interaction**				
1	*LMO2_P794 LMO2_E148 IL10_P325*		0.5928	0.0059
2	*LMO2_P794 LMO2_E148 GSTM1_P266*		0.5820	0.0577
3	*LMO2_E148 IL10_P325 GSTM1_P266*		0.5834	0.0059
4	*LMO2_P794 IL10_P325 GSTM1_P266*		0.5657	0.2517

* *The ranking was determined from the ratio of correct classifications to the total number of instances classified within the training dataset according to the function of track top models*.

# *The balanced accuracy was calculated across all cross-validation intervals as (Sensitivity + Specificity)/2*.

§ *The classification of high- and low-risk groups in two- or three-way-mode MDR analyses was validated through Chi-squared tests using the dataset from 361 subjects*.

## Discussion

The present study demonstrates that prenatal PTS exposure is associated with childhood asthma development at 6 years of age, but not associated with infant and children total IgE levels, or allergen sensitization at 6 years of age, implying that prenatal PTS-associated asthma is presumably mediated by an IgE-independent mechanism. Our study also shows that prenatal PTS exposure is associated with higher CG methylation levels of *LMO2* or *IL10,* and the combination of higher *LMO2_E148*, *IL10_P325* and *GSTM1_P266* methylation levels presented the highest risk of childhood asthma, 43.48%. This result highlights the fact that the development of childhood asthma is not limited to IgE-mediated mechanism but also prenatal PTS exposure associated with higher CG methylation of immune regulation genes in prenatal stage.

DNA methylation has long been implicated in gene dosage compensation for X-chromosome inactivation in females (Puck et al., 1990; Puck, 1993), and it is widely utilized for the prenatal diagnosis of X-linked immunodeficiencies (Puck et al., 1990; McCormack and Rabbitts, 2004). The DNA methylation levels of several genes have also been reported to significantly affect the risk of asthma (Morales et al., 2012; Runyon et al., 2012; Brunst et al., 2013; Kim et al., 2013; Naumova et al., 2013; Soto-Ramirez et al., 2013). Maternal tobacco smoke has been recently shown to program CG island methylation in certain signaling pathways including immune regulation pathway (Rotroff et al., 2016). In a genome-wide survey of CG methylation profiles in MTS reflected on cotinine levels (Ivorra et al., 2015; Rotroff et al., 2016), there are 15–25 genes linked to maternal cotinine-associated CG methylation changes, prominently (>90%), showing higher DNA methylation levels of the genes. The most striking CG methylation site was within the adrenomedullin gene in one report (Ivorra et al., 2015), and the other reported in the chromatin-binding complex and redox-regulation genes (*GFI1, MYO1G, CYP1A1, RUNX1, LCTL,* and *AHRR*) (Rotroff et al., 2016). These discordant findings of the DNA methylation in MTS exposure deserve further validation in different birth cohort studies. This may be because tobacco smoke-mediated DNA methylation is sex- and age-dependent DNA methylation (Breton et al., 2009; Rogers et al., 2009; Murphy et al., 2012; Naumova et al., 2013). We also found that *IL10_P325* CG methylation was significantly associated with prematurity in univariate and multivariate analyses, indicating CG methylation of *IL10* is regulated by perinatal conditions.

We are the first to show the higher CG methylation content of the *LMO2* or *IL10 is* correlated to prenatal exposure of PTS, and the combination of *LMO2_E148* (≧28.5%)*, IL10_P325* (≧38.5%), and *GSTM1_P266* (≧41.5%) reveals the highest risk to childhood asthma. The facts that *LMO2* is involved in lymphocyte differentiation and proliferation (Natkunam et al., 2007; Ryan et al., 2008), *IL10* is involved in immune regulatory functions (Hedrich et al., 2010), and *GSTM1* polymorphism is involved in tobacco-associated asthma (Gilliland et al., 2002; Wu et al., 2014), suggesting prenatal exposure of PTS could influence CG methylation in a series of genes involved in lymphocyte differentiation, immune regulation, and redox reaction. DNA methylation has been a long term biomarker of tobacco smoke exposure (Shenker et al., 2013). Preconception paternal smoking has been shown to alter sperm DNA methylation (Jenkins et al., 2017) and independently increased asthma risk in offspring (Accordini et al., 2018). Recently, three-generation cohort studies have shown that maternal grandmother’s smoking and maternal smoking during pregnancy (Braback et al., 2018), but not paternal grandmother’s smoking or paternal smoking, increased asthma risk in offspring (Accordini et al., 2018). It remains to be determined if the PTS-dependent CG methylation sites in the offspring is originated in prenatally secondhand tobacco exposure or in the germline imprint of male sperms.

There are some limitations in this study. First, not all children who completed the 6-year follow-up had DNA samples qualified for DNA bisulfite and pyrosequencing, reducing the study power and making selection bias possible. Next, we measured the CG methylation levels of whole leukocytes from umbilical cord blood, rather than from specific cell populations. Analysis of the whole blood cell population and specific subpopulations might reveal differences in the measured CG methylation levels. Further, only 1,505 CpG sites of Illumina GoldenGate array were surveyed, and only a couple of genes’ CpG sites were chosen for the validation by pyrosequencing experiments in the study. Finally, further studies to investigate the mechanisms involving the influence of *LMO2, IL10*, and *GSTM1* genes upon PTS and childhood asthma development are needed.

Literature has previously shown that offspring secondhand smoke exposure was associated with an increase in admission of respiratory diseases by 2 years of age (Snodgrass et al., 2016). We found that PTS was associated with infant frequent URIs, but the infants with frequent URIs were not significantly associated with CG methylation level of *LMO2, GSTM1*, or *IL10* in umbilical cord blood DNA. Whether the infants with frequent URIs associated with secondhand tobacco exposure is also related to methylation of certain CG sites beyond the genes we studied remains to be determined. We also found that the PTS-associated *LMO2_P794* and *GSTM1_P266* CG methylation contents in cord blood DNA significantly retained into those in children at age of 6. Taken together, these results suggest that PTS exposure affects CG methylation of immune regulation genes in prenatal stage and maintained into childhood associated with development of asthma.

## Data Availability

The raw data supporting the conclusions of this manuscript will be made available by the authors, without undue reservation, to any qualified researcher.

## Ethics Statement

This study was approved by the institutional review board of the study hospital, and the study informed consent was obtained from mother or father of the infant before study.

## Author Contributions

This birth cohort has proceeded for more than 6 years. The authors cited in the article are involved in design, recruitment of participants, patient care and follow up, as well as analysis. C-CW helped with patient care (children) and follow up, analysis, manuscript writing, and ascertainment of the cohort data. T-YH was a co-PI responsible for recruitment of participants (pregnant women), patient care, and informed consent (parents). J-CC helped with data coding, discussion, analysis of the cohort data. C-YO was a co-PI responsible for recruitment of participants (pregnant women), patient care, and informed consent (parents). H-CK helped with patient care (children), data coding, and follow up. C-AL helped with participation of cohort design, patient care (children), and follow up. C-LW helped with participation of cohort design, patient care (children), and follow up. HC was the Chief Manager of cohort data, in charge of the storage of blood samples, and measurement of specific IgE levels. C-PC helped with the recruitment of participants (pregnant women), patient care, and informed consent (parents). KY helped with design and organization of the cohort study, patient care (children), follow up, and analysis.

### Conflict of Interest Statement

The authors declare that the research was conducted in the absence of any commercial or financial relationships that could be construed as a potential conflict of interest.
